# Temporary mycophenolate discontinuation and reintroduction in pediatric kidney transplantation: predictors and long-term outcomes

**DOI:** 10.1007/s00467-026-07231-8

**Published:** 2026-04-14

**Authors:** Moran Plonsky Toder, Shirley Pollack, Rami Tibi, Irina Libinson-Zebegret, Renata Yakubov, Daniella Magen

**Affiliations:** 1 Technion - Israel Institute of Technology, The Ruth and Bruce Rappaport Faculty of Medicine, Haifa, Israel; 2 Rambam Health Care Campus, Ruth Rappaport Children’s Hospital, Pediatric Nephrology Institute, Haifa, Israel

**Keywords:** Graft survival, Immunosuppression reduction, Mycophenolate mofetil, Pediatric kidney transplantation, Opportunistic viral infections

## Abstract

**Background:**

Temporary discontinuation of mycophenolate mofetil (MMF) is frequently required in pediatric kidney transplant recipients, most often in response to viral complications. However, data regarding the long-term safety of MMF discontinuation, feasibility of reintroduction, and predictors of the need for MMF withdrawal in children remain limited.

**Methods:**

We conducted a retrospective, single-center cohort study of pediatric kidney transplant recipients with longitudinal follow-up. Clinical, virologic, immunologic, and transplant-related data were collected. Outcomes included longitudinal estimated glomerular filtration rate (eGFR), biopsy-proven acute rejection, graft loss, de novo donor-specific antibody (dnDSA) development, unplanned hospitalization, and MMF reintroduction. Factors associated with MMF discontinuation were evaluated using multivariable regression analysis.

**Results:**

Among 65 pediatric kidney transplant recipients, 22 (33.8%) required temporary MMF discontinuation, most commonly within the first post-transplant year. Opportunistic viral infections accounted for the majority of MMF withdrawal events, with BK virus as the predominant indication. MMF reintroduction was feasible in most patients (15/22). Longitudinal eGFR trajectories, rates of rejection, graft loss, dnDSA development, and unplanned hospitalization did not differ between patients who discontinued MMF and those who maintained full therapy. Younger age at transplantation emerged as the sole independent predictor of the need for MMF discontinuation.

**Conclusions:**

In this real-world pediatric cohort, temporary MMF discontinuation, when clinically indicated, closely monitored, and followed by cautious reintroduction, was not associated with adverse long-term graft outcomes. Younger age at transplantation was associated with a higher likelihood of MMF discontinuation, supporting individualized immunosuppression strategies and proactive efforts to identify children at risk for viral-driven immunosuppression intolerance.

**Graphical Abstract:**

**Figure:**
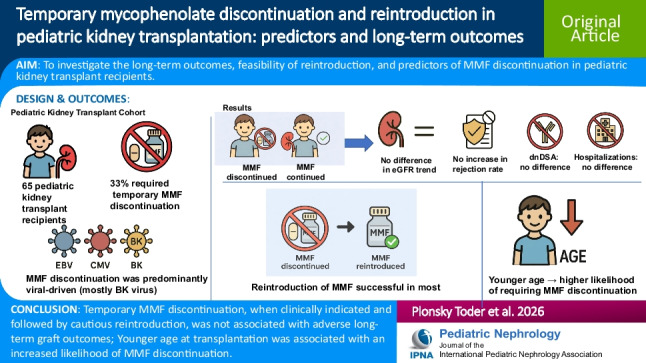
A higher resolution version of the Graphical abstract is available as Supplementary information.

**Supplementary Information:**

The online version contains supplementary material available at 10.1007/s00467-026-07231-8.

## Introduction

Long-term graft survival following pediatric kidney transplantation critically depends on sustained, yet appropriately balanced, immunosuppression. Achieving this balance remains challenging, as both allograft rejection and immunosuppression-related infectious complications contribute substantially to morbidity and mortality in this population [1, 2].

Mycophenolate mofetil (MMF) is a cornerstone of maintenance immunosuppression in pediatric kidney transplantation, typically administered in combination with a calcineurin inhibitor, with or without corticosteroids [3–6]. In routine clinical practice, however, immunosuppressive regimens frequently require modification in response to post-transplant complications, most notably opportunistic viral infections such as cytomegalovirus (CMV), Epstein–Barr virus (EBV), and BK virus, as well as leukopenia, gastrointestinal intolerance, or malignancy [2, 7]. Among maintenance agents, MMF is often the first to be modified, with approximately 30% of pediatric recipients requiring MMF discontinuation within the first two years following transplantation [7].

Despite the frequency of MMF modifications, high-quality evidence guiding MMF dose reduction, discontinuation, or reintroduction during post-transplant complications remains limited in both pediatric and adult literature [8, 9]. Adult studies suggest that MMF dose reduction beyond the first post-transplant year may be associated with reduced infectious complications without increasing rejection risk, suggesting that lower MMF exposure may be clinically acceptable in selected settings [9]. However, pediatric recipients differ substantially with respect to immune maturation, viral susceptibility, and rejection risk, limiting the applicability of adult data [2]. Previous pediatric studies demonstrate wide variability in the timing, indications, and management strategies of MMA discontinuation across centers and regions [2, 10, 11].

Our pediatric transplant cohort is characterized by high rates of consanguinity and uniform access to structured, long-term follow-up, providing a distinct clinical context in which to examine MMF modification strategies. This setting provides an opportunity to evaluate not only the clinical consequences of MMF discontinuation, but also patient- and transplant-related predictors associated with indications for MMF withdrawal. Despite its clinical relevance, data describing the frequency, determinants, and outcomes of MMF discontinuation, and the feasibility of subsequent reintroduction in pediatric kidney transplantation remain limited.

To address these gaps, we conducted a retrospective cohort study characterizing MMF discontinuation in pediatric kidney transplant recipients. Our primary objectives were to define the frequency, timing, duration, and indications for MMF withdrawal, identify clinical and transplant-related predictors of MMF discontinuation, assess the feasibility of MMF reintroduction, and evaluate long-term graft function and immunologic outcomes following MMF modification.

## Methods

### Study design and population

We conducted a single-center, retrospective cohort study including all pediatric kidney transplant recipients followed at the Pediatric Nephrology Institute, Rambam Health Care Campus, who underwent transplantation between January 1, 2012, and December 31, 2024. Follow-up continued until May 30, 2025. The study was approved by the Rambam Institutional Review Board (IRB 0254-24), with a waiver of informed consent due to pseudonymization of patient data.

Eligible patients were ≤ 21 years of age at the time of transplantation and had a minimum follow-up duration of 6 months. Patients transplanted at our center but followed elsewhere were excluded. Recipients of combined or sequential liver–kidney transplantation were included to reflect real-world pediatric transplant practice and were managed according to the same immunosuppression protocol as kidney-only recipients. For analytic consistency, only the first sustained MMF modification lasting > 2 weeks was recorded, using a pragmatic duration threshold similar to that applied in prior observational studies (e.g., [9]). Transient dose adjustments related to acute illness or drug interactions were excluded.

### Immunosuppression management and MMF modification

All patients received MMF or an equivalent dose of mycophenolic acid (MPA) as part of standard maintenance immunosuppression according to institutional protocol. MMF discontinuation was undertaken in response to clinically significant viral infection, leukopenia, gastrointestinal intolerance, or malignancy. In cases of persistent BK virus viremia after MMF withdrawal, leflunomide was considered as adjunctive immunomodulatory therapy.

MMF reintroduction was generally attempted following adequate control of viral infection or recovery of leukocyte count. Recurrent viral replication prompted renewed MMF discontinuation.

### Follow-up and data collection

Follow-up was defined from transplantation to graft loss, death, transfer to adult care, loss to follow-up, or study end. Data were manually extracted from electronic medical records, including demographic characteristics, transplant variables, immunosuppression exposure, virologic parameters, de novo donor-specific antibodies (dnDSA) development, rejection episodes, and clinical outcomes. When height was unavailable at specific time points, values were imputed using the last recorded height percentile to enable longitudinal glomerular filtration rate (eGFR) calculation, as previously described [12].

### Outcomes

Primary outcomes were longitudinal renal allograft function assessed by serial eGFR measurements, biopsy-proven acute rejection, and graft loss. Secondary outcomes included dnDSA development, clinically significant opportunistic viral infections (CMV, EBV, BK virus [4, 13]), feasibility of MMF reintroduction, subsequent MMF discontinuation events, unplanned hospitalization, and post-transplant malignancy.

### Statistical analysis

Statistical analyses were performed using R (version 4.5.1) and JASP (version 0.19.3). Continuous variables are presented as median (interquartile range [IQR]) or mean ± SD, as appropriate. Categorical variables were compared using *χ*^2^ or Fisher’s exact tests, and continuous variables using *t*-tests or Mann–Whitney *U* tests, as appropriate. Graft survival was analyzed using Kaplan–Meier curves and compared by log-rank testing. Longitudinal eGFR trajectories were analyzed using linear mixed-effects models. Variables with a *p *value < 0.1 in univariable analyses, or considered clinically relevant, were entered into Firth’s penalized logistic regression to identify factors associated with the need for MMF discontinuation. Statistical significance was defined as *p* < 0.05.

## Results

### Cohort characteristics

During the study period, 78 pediatric kidney transplant recipients were followed at our center. After exclusion of 12 patients without sufficient follow-up and one peri-transplant death, the final cohort comprised 65 patients (38.5% female), with a median age at transplantation of 9.66 years (IQR 5.2–15.9). Four patients (6.1%) underwent combined or sequential liver–kidney transplantation. Median follow-up duration was 4.1 years (IQR 1.75–6.9). Baseline demographic and clinical characteristics are summarized in Table 1.

**Table 1:** Demographic, clinical, and graft characteristics by MMF discontinuation status

	All kidney recipients (*N* = 65)	MMF discontinuation (*N* = 22)	No MMF discontinuation (*N* = 43)	*p* value
Age at transplant, y (median [IQR])	9.66 (5.2–15.9)	5.36 (3.57–7.63)	12.8 (6.57–16.4)	< 0.01
Sex, female *N* (%)	25 (38.5)	9 (40.9)	16 (37.2)	1
Religion *N* (%)				0.52
Muslim	43 (66.2)	13 (59.1)	30 (69.8)	
Jewish	14 (21.5)	5 (22.7)	9 (20.9)	
Other	8 (12.3)	4 (18.2)	4 (9.3)	
**Pre-transplant**				
* Dialysis modality*				0.13
Hemodialysis	55 (84.6)	19 (86.4)	36 (83.7)	
Peritoneal dialysis	5 (7.7)	3 (13.6)	2 (4.7)	
Pre-emptive transplantation	5 (7.7)	0 (0)	5 (11.6)	
* Etiology of kidney failure N (%)*				0.55
Immune disease	16 (24.62)	4 (18.2)	12 (27.9)	
Non-immune disease	49 (75.38)	18 (81.8)	31 (72.1)	
**Transplant-related characteristics**				
* Donor type N (%)*				0.55
Deceased	41 (63.1)	16 (72.7)	25 (58.1)	
Living related	20 (30.7)	5 (22.7)	15 (34.9)	
Living non-related	4 (6.2)	1 (4.6)	3 (7)	
* HLA mismatch median (IQR)*	5 (3–6)	5 (3.25–6)	5 (3–6)	0.63
* Induction therapy N (%)*				1
Anti-IL2 + corticosteroids	59 (90.8)	20 (90.9)	39 (90.7)	
ATG + corticosteroids	6 (9.2)	2 (9.1)	4 (9.3)	
* Maintenance immunosuppression N (%)*				0.33
Tac + MMF + steroids	64 (98.5)	21 (94.4)	43 (100)	
mTORi + MMF + steroids	1 (1.5)	1 (4.6)	0 (0)	
* Tacrolimus level (ng/ml) mean, (S*D)				
1 st month post-transplant	11.43 (2.165)	10.92 (2.3)	11.69 (2.07)	0.23
2nd month post-transplant	11.16 (2.144)	10.55 (2.3)	11.46 (2.02)	0.09
≥ 3rd month post-transplant	7.46 (0.89)	7.27 (0.68)	7.55 (0.97)	0.77
Length of follow-up, y (median [IQR])	4.1 (1.75–6.9))	3.97 (1.58–6.97)	4.22 (2.47–6.4)	0.94

Abbreviations: *N*, number; *MMF*, mycophenolate mofetil; y, years; *IQR*, interquartile range; *HLA*, human leukocyte antigen; *ATG*, anti-thymocyte globulin; *IL-2*, interleukin-2; *mTORi*, mammalian target of rapamycin inhibitor; *Tac*, tacrolimus; *SD*, standard deviation

Twenty-two patients (33.8%) required MMF discontinuation. Among these patients, the median duration of MMF interruption was 4.1 months (IQR 3.0–18.0), with 17 patients (77.3%) experiencing interruptions lasting ≥ 2 months and lasting longer than 2 weeks. The predominant indication was opportunistic viral infection (19/22, 86.4%), followed by leukopenia (2/22, 9.1%) and gastrointestinal intolerance (1/22, 4.5%). Median time from transplantation to MMF discontinuation was 7.6 months (IQR 2.2–13.4). Induction regimen, tacrolimus trough levels, and corticosteroid exposure were comparable between patients who discontinued MMF and those who maintained full therapy. Seven patients remained off MMF at the end of follow-up due to intolerance of reintroduction.

### Primary outcomes

#### Renal allograft function

**Fig. 1:**
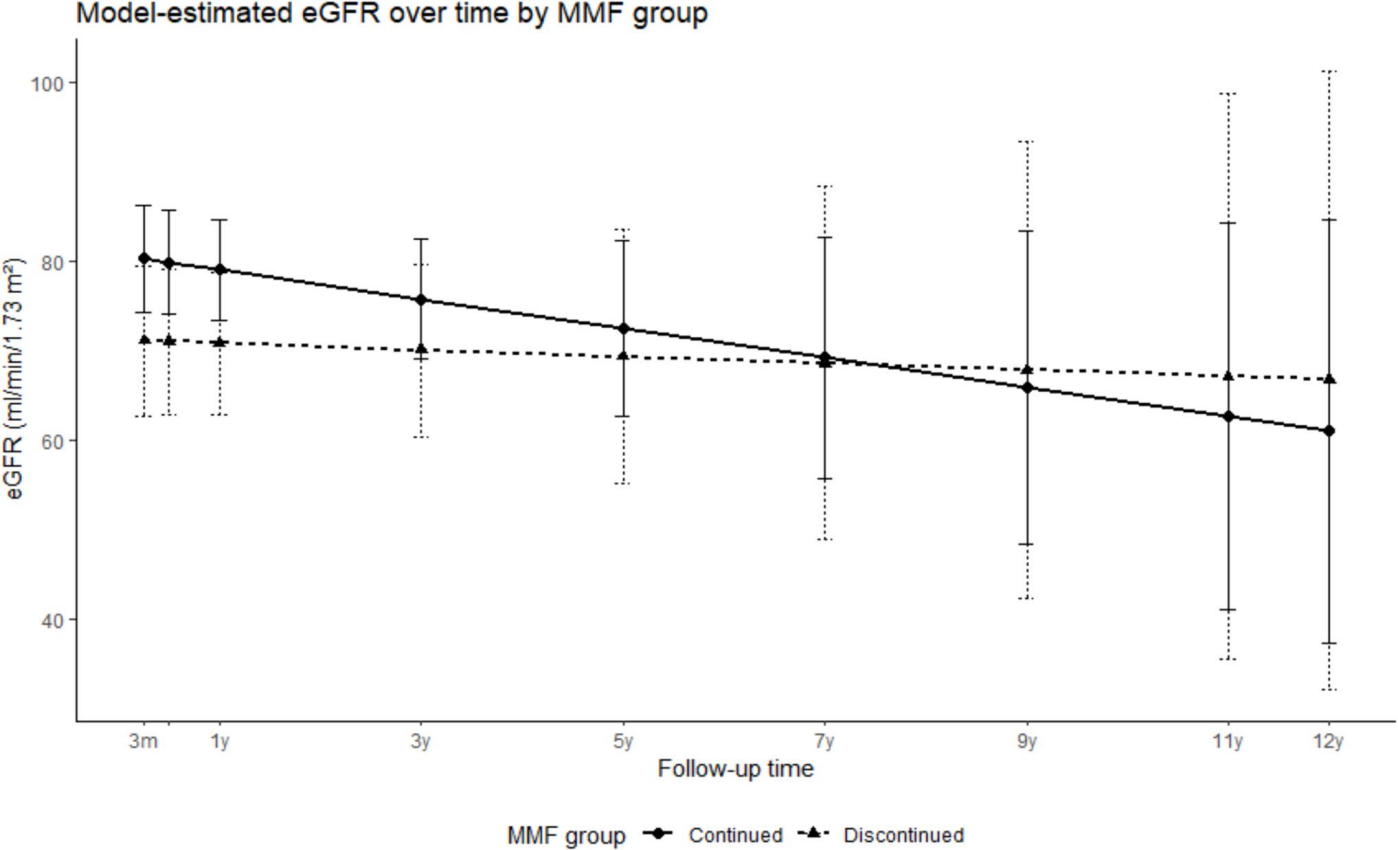
Model-estimated longitudinal changes in estimated glomerular filtration rate (eGFR) in pediatric kidney transplant recipients who either maintained mycophenolate mofetil (MMF) therapy or experienced MMF discontinuation during follow-up. Points represent estimated marginal means derived from a linear mixed-effects model; error bars indicate 95% confidence intervals. No significant difference in long-term eGFR slope was observed between groups

In linear mixed-effects model evaluating longitudinal eGFR, MMF discontinuation was not associated with lower eGFR over time (group effect* p* = 0.08), and the rate of eGFR decline did not differ between groups (time × group interaction *p* = 0.5). Model-estimated eGFR trajectories are shown in Fig. 1.

#### Biopsy-proven acute rejection

Over 306 patient-years of follow-up, 10 biopsy-proven rejection episodes occurred (3.27 episodes per 100 patient-years), including six cellular, three antibody-mediated, and one mixed cellular and antibody-mediated rejection. Nine episodes occurred in the control group and one in the MMF discontinuation group, with no statistically significant difference between groups (*p* = 0.08).

#### Graft loss

**Fig. 2:**
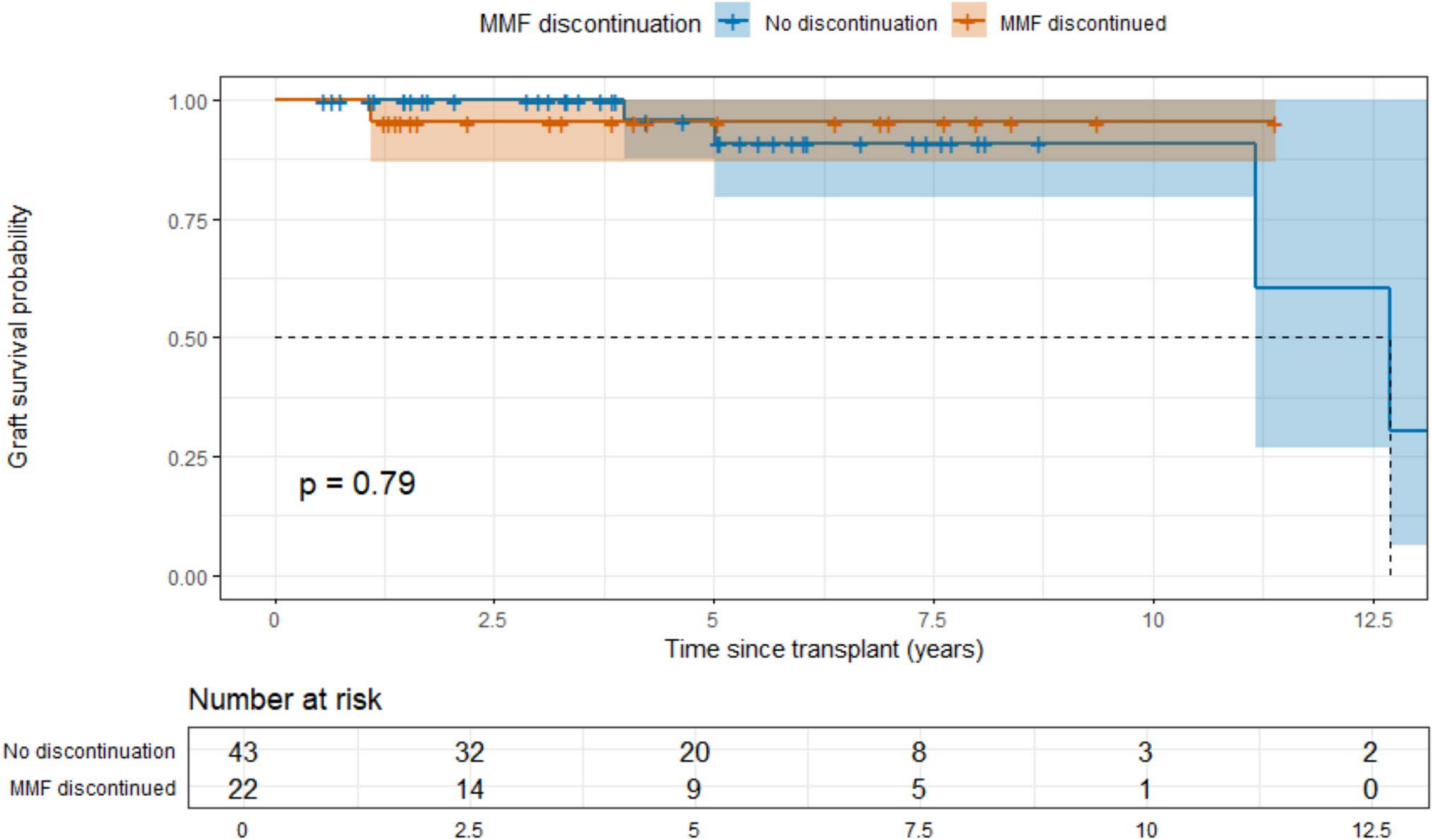
Kaplan–Meier curves depicting time from kidney transplantation to graft failure in pediatric kidney transplant recipients who discontinued mycophenolate mofetil (MMF) during follow-up, compared with those who remained on continuous MMF therapy. Patients with a functioning graft at the last follow-up were censored. No significant difference in graft survival was observed between groups (log-rank test, *p* = 0.79). Numbers at risk are shown below the x-axis

Five graft losses occurred during follow-up at a mean of 8.0 ± 4.4 years post-transplant. Only one graft loss occurred in the MMF discontinuation group, with no difference in graft survival compared with controls (*p* = 0.7; Fig. 2). Overall, no significant differences were observed between groups with respect to eGFR trajectories, rejection episodes, or graft survival.

### Secondary outcomes

#### De novo donor-specific antibodies (dnDSA)

Valid dnDSA data were available for 56 patients. Results were missing for eight patients in the control group and one in the MMF discontinuation group, maintaining a similar proportional distribution. dnDSA developed in 12 patients (9 controls, 3 MMF discontinuation), with no difference between groups (*p* = 0.5).

#### Opportunistic viral infections

During follow-up, 31 patients (47.7%) experienced at least one opportunistic viral infection, most commonly BK virus (35.4%), followed by CMV (20%) and EBV (13.8%). Nearly all patients in the MMF discontinuation group developed a viral infection (20/22, 90.9%), compared with significantly fewer in the control group (95% CI 1.67–5.62, *p* < 0.01). Pre-transplant viral exposure did not differ significantly between groups.

#### Hospitalizations

The median rate of unplanned hospitalizations lasting ≥ 24 h was 1.49 events per patient-year (IQR 0.87–2.58) and did not differ between patients who discontinued MMF and those who did not (1.33 vs. 1.56 events per patient-year, respectively, *p* = 0.90).

#### MMF reintroduction

MMF was successfully reintroduced in 15 of 22 patients (68.2%). Seven patients remained off MMF due to persistent or recurrent viral infection or prior PTLD. Failure of MMF reintroduction was commonly associated with persistent or recurrent BK virus viremia, including cases in which viremia recurred upon MMF rechallenge after initial viral clearance. Patients unable to tolerate reintroduction had a significantly longer cumulative duration off MMF compared with those who resumed therapy (median 30.5 vs. 4.1 months, respectively, *p* < 0.01), but eGFR at last follow-up did not differ between groups. Characteristics of this subgroup are summarized in Table 2.

**Table 2:** Demographic and clinical characteristics of patients for whom MMF reintroduction was not feasible at the end of follow-up

No	Age at transplant (y)	Etiology of kidney failure	Cause of MMF discontinuation	MMF course (y) time to d/c/duration off	Cause of failed reintroduction	dnDSA	Graft function at last f/u (eGFR)	Other complications
1	3.14	s/p bilateral nephrectomy- Wilms tumor (no *WT1* mutation)	BKV	0.65/5.98	Persistent or recurrent BKV viremia	Neg	Functioning	TCC of bladder- BKV associated (appeared while off MMF)
2	3.53	CNS (nephrin)	BKV	1.03/5.88	Persistent or recurrent BKV viremia	Pos	Functioning	
3	2.52	Renal-diabetes syndrome	BKV	0.63/5.75	Multiple viral infections	Neg	Functioning	
4	5.71	CNS (CRB2)	PTLD and CMV	1.3/2.54	PTLD in 1 st year	Pos	Functioning	PTLD
5	7.88	CAKUT	BKV	1.54	Persistent or recurrent BKV viremia	Neg	Functioning	
6	3.98	Takayasu arteritis	BKV	0.16/1.28	Persistent or recurrent BKV viremia	Neg	Functioning	
7	15.36	Lupus nephrites	BKV	0.16/1.28	Persistent or recurrent BKV viremia	Neg	Functioning	

Abbreviations: *No.*, number; *MMF*, mycophenolate mofetil; *d/c*, discontinuation; *dnDSA*, de novo donor-specific antibodies; *f/u*, follow-up; *eGFR*, estimated glomerular filtration rate; *s/p*, status post; *WT1*, Wilms tumor suppressor gene 1; *CNS*, congenital nephrotic syndrome; *BKV*, BK virus; *CMV*, cytomegalovirus; *PTLD*, post-transplant lymphoproliferative disorder; *CAKUT*, congenital anomalies of the kidney and urinary tract; *TCC*, transitional cell carcinoma; *Neg*, negative; *Pos*, positive; *y*, years

#### Malignancy

Three malignancies occurred during follow-up, including one early PTLD, one cutaneous pre-lymphomatous disease, and one of BK virus-associated transitional cell carcinoma of the bladder. All patients responded to MMF discontinuation and specific oncologic therapy, without graft loss.

#### Predictors of the need for MMF discontinuation

On univariate analysis, baseline characteristics were similar between groups except for age at transplantation. In Firth’s penalized logistic regression, younger age at transplantation emerged as the only independent predictor of the need for MMF discontinuation (OR = 0.83, 95% CI 0.72–0.94, *p* = 0.002). Donor type, etiology of primary kidney disease, and pre-transplant CMV or EBV exposure were not associated with MMF discontinuation. The multicollinearity test did not reveal any collinearity with VIF < 2 for all included variables. Regression results are shown in Table 3.

**Table 3:** Multivariable Firth logistic regression identifying factors associated with MMF discontinuation

Variable	OR	CI	*p* value	sig
(Intercept)	1.91	0.35–10.71	0.45	
**Age at transplant (y)**	**0.83**	**0.72–0.94**	**0.002**	**
Donor-recipient related	1.96	0.57–7.5	0.29	
Pretransplant viral exposure	0.78	0.19–3.32	0.73	
Diagnosis: immunologic vs. non-immunologic	1.93	0.42–9.4	0.4	

Abbreviations: *y*, years; vs, versus; *OR*, odds ratio; *CI*, confidence interval; *sig*, significance

## Discussion

In this retrospective single-center cohort, we examined the frequency, indications, and clinical consequences of MMF discontinuation in pediatric kidney transplant recipients. Approximately one-third of patients required temporary MMF discontinuation, most commonly within the first year post-transplant, reflecting clinician-directed, indication-driven decisions made in response to post-transplant complications. Opportunistic viral infections were by far the predominant trigger for MMF withdrawal, whereas leukopenia and gastrointestinal intolerance were relatively infrequent indications. The frequency and distribution of MMF discontinuation in our cohort were comparable to those reported in prior pediatric series [7, 10, 11]. Aside from selective use of leflunomide for BK virus viremia, MMF withdrawal was not accompanied by routine escalation of alternative immunomodulatory therapies, and background immunosuppression including corticosteroid exposure and tacrolimus levels remained comparable between patients who discontinued MMF and those who did not.

Opportunistic viral infections—predominantly EBV, CMV, and BK virus—are well-recognized markers of over-immunosuppression and commonly prompt immunosuppression reduction to facilitate viral clearance, albeit at the potential cost of increased alloimmune risk [6, 13, 14]. In our cohort, BK virus was the most frequent infection necessitating MMF withdrawal and the principal reason for failure of MMF reintroduction. While pre-transplant EBV and CMV serostatus did not predict subsequent MMF discontinuation, assessment of baseline BK virus risk remains challenging, as no clinically available assay exists. Most pediatric BK virus infections are thought to arise from donor-derived latent virus in the uroepithelium, particularly in seronegative recipients [14–16]. In this context, early recognition of excessive immunosuppression and timely MMF reduction may represent a protective strategy against BK virus-associated graft injury.

Despite variability in the literature regarding the impact of MMF interruption on graft outcomes [7–10], we observed no significant long-term differences in renal allograft function trajectories between children who discontinued MMF and those who maintained standard triple immunosuppression. Rates of biopsy-proven acute rejection, graft survival, and dnDSA appearance were comparable between groups. Together, these findings suggest that temporary MMF discontinuation, when clinically indicated, closely monitored, and followed by reintroduction when feasible, may not necessarily compromise long-term allograft outcomes. In our cohort, MMF withdrawal was typically time-limited and guided by clinical or virologic recovery, with preservation of calcineurin inhibitor and corticosteroid exposure, which may have mitigated alloimmune risk during periods of MMF interruption.

Rates of unplanned hospitalization were comparable between patients who discontinued MMF and those who maintained full therapy, despite a higher incidence of opportunistic viral infections in the former group, raising the possibility that timely MMF reduction may mitigate the clinical impact of viral complications.

MMF reintroduction was attempted after resolution of the precipitating complications and was successfully tolerated in the majority of cases, supporting the feasibility of cautious MMF reinstatement in selected pediatric recipients. Moreover, in our center, MMF reintroduction is typically gradual, requiring 2–4 weeks to reach full dosing; thus, even relatively short periods of complete MMF withdrawal may be associated with a longer overall duration of reduced immunosuppression.

A subset of patients was unable to tolerate reintroduction, most commonly due to persistent BK virus viremia, resulting in a longer cumulative duration off MMF. Despite prolonged MMF interruption, long-term graft outcomes in this subgroup did not differ from those observed in patients who successfully resumed therapy. Two malignancies occurred among patients in this subgroup, including one early PTLD and one BK virus-associated transitional cell carcinoma of the bladder diagnosed several years after MMF discontinuation. Given the small number of events, these observations should be interpreted cautiously and viewed as hypothesis-generating. Whether the inability to tolerate MMF reintroduction reflects underlying host susceptibility or is primarily driven by persistent viral replication and cumulative immunosuppressive exposure warrants further study.

In exploring predictors of the need for MMF discontinuation, younger age at transplantation emerged as the sole independent factor associated. Traditional clinical variables—including donor type, underlying disease etiology, and pre-transplant viral exposure (CMV or EBV)—were not predictive. This finding aligns with prior pediatric studies suggesting an age-dependent vulnerability to requiring MMF modification [7, 11], while extending these observations by demonstrating a significant independent association. Together, these observations reinforce younger age as a clinically meaningful marker of susceptibility to immunosuppression-related complications.

Several age-related factors may underlie this association. Clinically, younger pediatric kidney transplant recipients are more likely to experience primary viral infections under immunosuppression, reflecting limited prior viral exposure and an increased likelihood of early post-transplant infections [14, 17]. This heightened viral susceptibility may predispose younger children to viral-driven immunosuppression intolerance and necessitate more frequent MMF modification. From a biological perspective, early life is characterized by a predominance of naïve adaptive immune cells and ongoing maturation of both B- and T-cell repertoires, resulting in limited pre-existing antiviral immunity and reduced capacity to generate durable memory responses compared to older children and adults [18, 19]. Under immunosuppressive therapy, this developmental immaturity may further impair viral control and contribute to persistent or recurrent viral replication. In addition, age-dependent differences in immune reconstitution and immunosuppressive drug handling may further influence susceptibility to infection and tolerance of maintenance immunosuppression [17]. Importantly, the lack of association between pre-transplant viral serostatus and MMF discontinuation in our cohort underscores the limitations of current clinical markers in identifying children at risk for viral-driven immunosuppression intolerance.

From a clinical perspective, these findings support a more individualized approach to immunosuppression management in pediatric kidney transplant recipients, particularly among younger children. This highlights the importance of balancing infectious risk and alloimmune protection when modifying maintenance immunosuppression in response to post-transplant complications.

This study has several limitations. Its retrospective, single-center design and relatively small sample size may limit generalizability, although the use of uniform immunosuppressive protocols and structured follow-up at our institution enhances internal consistency. MMF discontinuation represents a time-dependent exposure; however, given the limited cohort size and number of outcome events, patients were analyzed according to whether MMF discontinuation occurred at any point during follow-up. The study was not powered to detect small differences in relatively infrequent outcomes such as biopsy-proven rejection or graft loss, and follow-up duration varied across patients. Pre-transplant BK virus serostatus was unavailable, precluding assessment of baseline viral risk, and routine therapeutic drug monitoring of mycophenolate exposure was not performed, limiting our ability to evaluate the dose-exposure relationship. Although missing data were minimal, they may have introduced minor imprecision in longitudinal analyses.

Future efforts should focus on improving the identification of children at risk for viral-driven immunosuppression intolerance before clinical complications arise. Emerging tools such as torque teno virus (TTV) load, donor-derived cell-free DNA, and immunophenotyping hold promise for guiding immunosuppression management, although these approaches are not yet routinely available. Integrating longitudinal clinical data with immunologic and genetic markers may ultimately enable more precise, anticipatory immunosuppression strategies in pediatric kidney transplantation.

In this pediatric kidney transplant cohort, temporary MMF discontinuation was common and most frequently driven by opportunistic viral infections. When guided by clinical and virologic recovery and followed by cautious reintroduction, MMF modification was not associated with adverse long-term graft or immunologic outcomes. Younger age at transplantation emerged as a key predictor of the need for MMF discontinuation, underscoring age-related vulnerability to immunosuppression intolerance and the importance of individualized immunosuppressive strategies.

## Supplementary Information

Below is the link to the electronic supplementary material.

Graphical abstract (PPTX 4.42 MB)

## Data Availability

The data that support the findings of this study are available from the corresponding author upon reasonable request.
