# Supplementary material for: Temporary mycophenolate discontinuation and reintroduction in pediatric kidney transplantation: predictors and long-term outcomes
Source: Pediatr Nephrol. 2026 Apr 14;41(9):3059–67. doi: 10.1007/s00467-026-07231-8 (PMC13424366; doi:10.1007/s00467-026-07231-8)
Supplement: Supplementary file 1 — Graphical abstract (PPTX 4.42 MB) [file 467_2026_7231_MOESM1_ESM.pptx]

## Slide 1
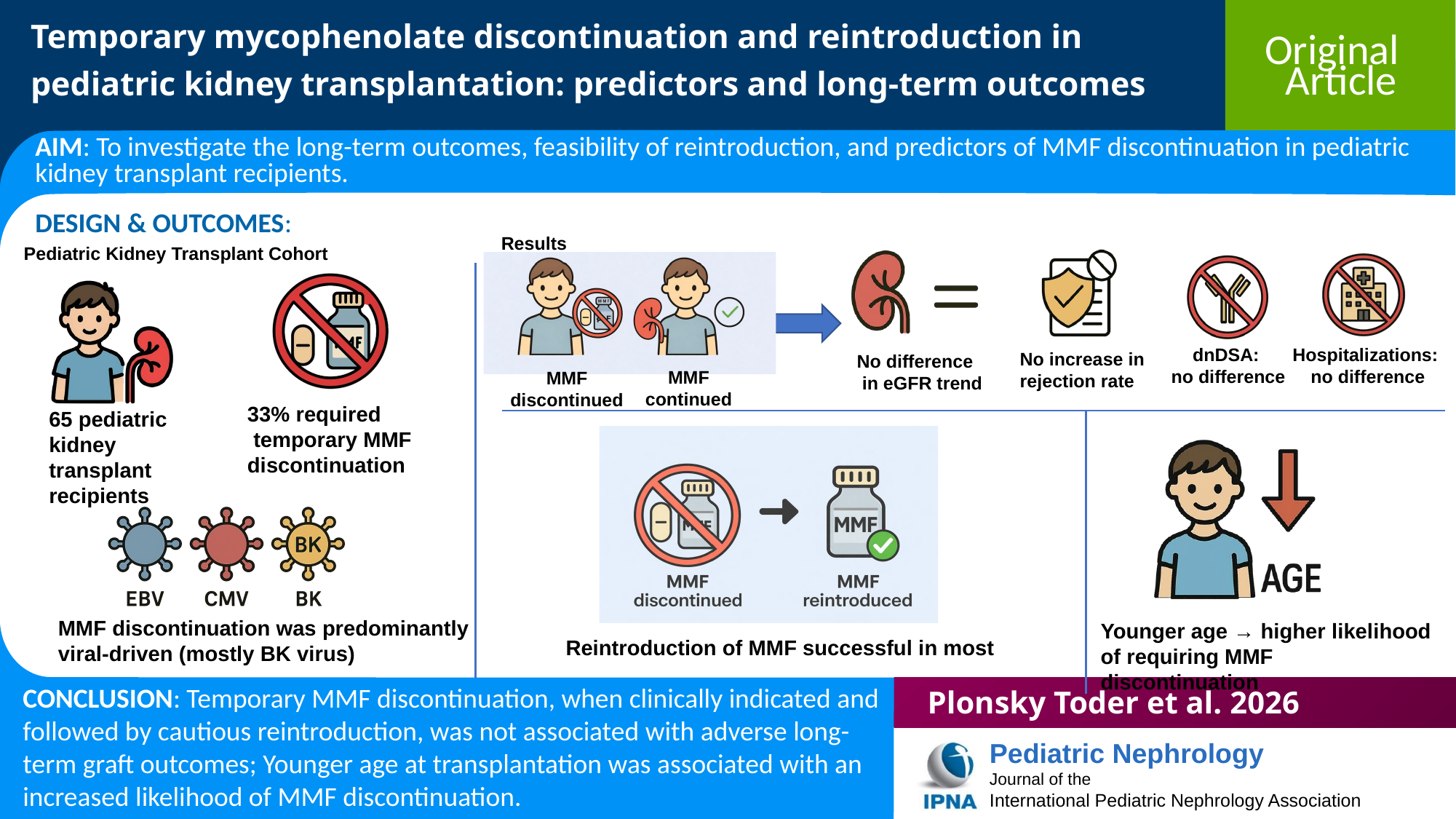

Temporary mycophenolate discontinuation and reintroduction in pediatric kidney transplantation: predictors and long-term outcomes
AIM: To investigate the long-term outcomes, feasibility of reintroduction, and predictors of MMF discontinuation in pediatric kidney transplant recipients.
DESIGN & OUTCOMES:
Results
Pediatric Kidney Transplant Cohort
Hospitalizations:
 no difference
dnDSA:
 no difference
No increase in rejection rate
No difference
 in eGFR trend
MMF continued
MMF discontinued
33% required
 temporary MMF
discontinuation
65 pediatric kidney
transplant recipients
MMF discontinuation was predominantly viral-driven (mostly BK virus)
Younger age → higher likelihood of requiring MMF discontinuation
Reintroduction of MMF successful in most
CONCLUSION: Temporary MMF discontinuation, when clinically indicated and followed by cautious reintroduction, was not associated with adverse long-term graft outcomes; Younger age at transplantation was associated with an increased likelihood of MMF discontinuation.
Plonsky Toder et al. 2026
